# A systematic integrative review of programmes addressing the social care needs of older prisoners

**DOI:** 10.1186/s40352-019-0090-0

**Published:** 2019-05-27

**Authors:** Caroline Lee, Samantha Treacy, Anna Haggith, Nuwan Darshana Wickramasinghe, Frances Cater, Isla Kuhn, Tine Van Bortel

**Affiliations:** 1 Cambridge Institute of Public Health, University of Cambridge, Cambridge, UK; 2 Department of Community Medicine, Faculty of Medicine and Allied Science, Rajarata University of Sri Lanka, Saliyapura, Sri Lanka; 3 Medical Library, University of Cambridge, Cambridge, UK

**Keywords:** Prison, Older prisoners, Social care, Social work, Hospice, Prisoner peer support, Systematic review, Integrative review

## Abstract

**Background:**

The number of older prisoners has risen exponentially over the last two decades, especially in high-income countries. Due to the increased and somewhat inadequately met health and social care needs of this group of prisoners, coupled with their vulnerability arising from higher levels of isolation, poverty and exploitation, financial costs have spiralled and human rights concerns have grown. This review aimed to present an overview of programmes that addressed older prisoners’ social care needs, a particularly underdeveloped area, with a view to assessing the extent to which they could inform policy and practice.

**Methods:**

Following Whittemore and Knafl’s (J Adv Nurs 52:546-553, 2005) integrative review approach, a comprehensive search - including 16 electronic databases and hand searching - was undertaken up to May 2017 using search terms related to context, function and disability. The quality of included papers was assessed, data were extracted using a review-specific form based on the PICO formula, and research questions addressed using a narrative synthesis approach. Additionally, reporting followed PRISMA guidelines.

**Results:**

A total of 29 papers were selected for inclusion, the majority of which focused on hospice programmes, with the remainder describing personal care-focused services, structured day programmes, and adaptations to prison operations (regime) and accommodation in support of prisoners’ social care needs. Whilst the programmes were reported to have some positive impacts on prisoners and the prison overall, and programmes were perceived to be cost-effective or cost-neutral, outcomes regarding staff were more mixed. Findings were tempered by the methodological shortcomings of the included papers, with many assessed as low quality, with a lack of prisoner participation, and an absence of experimental studies.

**Conclusions:**

The evidence base for programmes addressing older prisoners’ social care needs appears to be at an embryonic stage. Further robust studies evaluating the effectiveness and cost-effectiveness of programmes addressing older prisoners’ social care needs are imperative in better informing policy and practice in support of this highly vulnerable group.

**Electronic supplementary material:**

The online version of this article (10.1186/s40352-019-0090-0) contains supplementary material, which is available to authorized users.

## Background

In many high-income countries, the population of older prisoners (defined here as those over the age of 50^1^) is growing faster than any other age group (Atabay, 2009; Enggist, Møller, Galea, & Udesen, 2014). For example, older prisoner numbers have more than tripled in the USA, Japan, and England and Wales over the last couple of decades and are growing (Allen & Watson, 2017; Carson & Sabol, 2016; Ministry of Justice, 2018a; The Ministry of Justice, 2017). This increase has been attributed to the general ageing of populations, and to harsher and longer prison sentencing policies (Atabay, 2009; Hantke, Bretschneider, Elgar, & Wangmo, 2017). More local contributions include increases in the prosecution of historic sex offences in the UK and Australia (Justice Committee, 2013; O’Brien, Tewaniti, Hawley, & Fleming, 2006), and increases in petty crime in Japan and South Korea due to poverty (Allen, 2016; Kamigaki & Yokotani, 2014). The growth rate of older prisoners in lower-income countries appears to be more varied (Atabay, 2009; Chitsawang, 2017; Department of Prisons, 2017; Langat, Kabaji, & Poipoi, 2015; Srinivasan & Ponnuswami, 2015).

Concerns have been expressed about the spiralling costs of imprisoning large numbers of older people. These are estimated to be three times that of younger prisoners largely due to increased health and social care needs (Bedard, Metzger, & Williams, 2016; Senior et al., 2013), reportedly affecting around 85–90% of prisoners over the age of 50 (Di Lorito, Völlm & Dening, 2018; Hayes, Burns, Turnbull, & Shaw, 2012; Senior et al., 2013). It has become an international policy norm that prisons provide a standard of care equivalent to that of the community (United Nations, 1990). However, the quality of prison healthcare is thought to vary across the world, partly due to resources, and even in higher income countries it has been reported as patchy or inadequate (Enggist et al., 2014; Health and Social Care Committee, 2018a; Jotterand & Wangmo, 2014). The provision of prison social care has been described in even more parlous terms, with infrequent to non-existent social care contact for prisoners, exacerbated by unclear lines of responsibility (Justice Committee, 2013; Pettus-Davis, 2012; Scheyett, Pettus-Davis, McCarter, & Brigham, 2012; Scottish Prison Service, 2017).

Whilst the line between health and social care is porous, this review defines social care needs as: “needing regular help looking after oneself because of illness, disability or old age” (Bottery, Varrow, Thorlby, & Wellings, 2018, p 29), alleviation of social isolation and maintenance of independence (Department of Health, 2012), as well as hospice and palliative care (Hughes, Firth, & Oliviere, 2014). This is in contrast to the provision of healthcare treatment-focused interventions such as medication or psychotherapy. Older prisoners’ social care difficulties reportedly include: functional and mobility impairments (for example, difficulties with bathing facilities, problems with collecting meals or climbing stairs to reach activities) and increased social isolation (where regimes keep retired or disabled prisoners locked in their cells if they do not work) (Enggist et al., 2014; Hayes et al., 2012; Hayes, Burns, Turnbull, & Shaw, 2013; Joyce & Maschi, 2016; Snyder, van Wormer, Chadha, & Jaggers, 2009). In addition, older prisoners social care needs may be impacted by: loss of family contact (with increased likelihood of bereavement and visiting difficulties), bullying by younger prisoners, prison poverty (with less access to employment or family help), poor availability of appropriate activities (employment or gym sessions that are too physically demanding), and inadequate resettlement assistance (especially securing accommodation for release) (Aday & Farney, 2014; Cornish, Edgar, Hewson, & Ware, 2016; Hayes et al., 2012, 2013; Joyce & Maschi, 2016; Snyder et al., 2009).

The literature has broadly defined four key ways in which older prisoners’ social care needs are or could be met. These include (i) adaptations to prison environments and systems, such as separate wings to safeguard from bullying, stair lifts to aid mobility or allowing non-working prisoners out of their cells through the day to reduce isolation (Lee et al., 2016); (ii) personal care - focused on assisting prisoners with their activities of daily living [ADLs] (Lee et al., 2016), (iii) structured day programmes (Stevens et al., 2017) which focus on the ADLs, activities and social needs of older prisoners generally, or those with more specific conditions such as dementia; and (iv) hospices which attend to dying prisoners’ ADLs, family and social care needs, often involving social workers in their development and management (Bronstein & Wright, 2007).

There is a dearth of research and evaluations of social care practice in prisons generally, and for older prisoners specifically, with a lack of overarching programmes, models or guidelines (Senior et al., 2013; Tucker et al., 2017), and no systematic reviews found to support development of the field. A systematic review of older prisoner ‘care’ interventions only described two papers which supported prisoners social care needs through structured day programmes (Stevens et al., 2017). It has been suggested that a lack of access to social care for older prisoners potentially breaches equalities and human rights legislation (Lee et al., 2016; Williams, 2013), and partly triggered a parliamentary inquiry in one higher income country (Health and Social Care Committee, 2018b). It is the intention of this paper to comprehensively review the existing evidence base of programmes which support the social care needs of older prisoners, in order to explore the extent to which they can inform policy and practice, and to identify directions for future research to better inform their evolution.

## Research questions

In order to meet the overall aims of this review, the following questions were postulated:

1. What types of programmes were described in the research to address the social care needs of older prisoners?
2. What methods have been used in the reporting of programmes or interventions which support the social care needs of older prisoners, and what is the quality of that research?
3. What were the reported outcomes of programmes addressing older prisoners’ social care needs?

## Methods

Given the scarcity of research in this area, a brief pilot search was conducted upon which it was decided to employ a systematic integrative review methodology. This method offered the flexibility needed to incorporate a wide variety of study methodologies and provided a systematic approach to conducting the literature review, enabling us to meet the aims of the research. This particular integrative review primarily used an adapted version of Whittemore and Knafl’s (2005) approach, with reporting informed by PRISMA systematic review guidelines and checklist (Moher et al., 2009) - see Additional file 1 for the completed PRISMA 2009 checklist for this review. Following this process, four stages were completed for the review:

### Literature search

The search strategy was formulated by the research team with a senior librarian, and refined by pilot searches. Systematic and iterative search techniques were used with 16 electronic databases related to clinical and social sciences, without date restriction. These were Medline, Embase, PsycINFO, CINAHL, Web of Science, SCIE, Cochrane Library, Campbell Collaboration, Sociological Abstracts, DARE, ASSIA, Social Services Abstracts, National Criminal Justice Reference Service, DoPHER, TRoPHI, and Health Evidence Canada. Search terms were split into three categories encompassing: (i) status/context, (ii) support mechanism/functionality, and (iii) condition/age-related disability as given in Table 1 – free text and appropriate subject headings were used where possible. A search was then carried out in each database combining context/status AND support/functionality AND condition/age-related disability.

**Table 1:** Indicative search terms used in literature search

Status/context	Support mechanism/functionality	Condition/Age related disability
prison* or convict* or felon* or offender* or inmate* or criminal* or jail* or penitentiar* or gaol* or secure or correctional	Nurs* or care or caring or support* or peer* or buddy* or buddies* or friend* or “cell mate*” or mentor* or be-friend* or befriend* or “lay person*” or volunteer* or voluntar* or insider or listener or “mobility disorder” or mobil* or “independent liv*” or “independent life*” or “activities of daily living” or “daily activities” or “daily life activity” or adl* or eadl* or dressing or feeding or eating or toilet* or bathing or “social*(support* or active* or function* or behav* or adjust* or skill*)” or facilitate* or “self care” or “self manage*” or “personal care” or “personal manage*”	frail elderly or frail* or chronic or disabilit* or disabled* age(“degenerate*disorder”) or dementia* or alzheimer* or cognitive defect or “cognition disorder” or parkinson* or mobility* or deaf* or “hearing los*” or “hearing disorder*” or “hearing impair*” or blind* or glaucoma or “macular degenerat*” or “vis* impair*” or “vis* disorder*” or “vis* reduc*” or “vision difficult*” or hearing, or eye or vision or blind or sight or blindness or comorbid* or co-morbid* or terminal* or palliative* or “right to die” or neoplasm or cancer*

The electronic database search was supplemented by reference mining and hand searching of selected journals and industry publications. The searches covered the full range of publications up to May 2017, published in all languages. An example search strategy is given in Additional file 2.

### Data evaluation

Papers identified by the search were screened by title, abstract and full-text by two independent reviewers, to check inter-rater reliability, as recommended by Social Care Institute for Excellence [SCIE] (Rutter, Francis, Coren, & Fisher, 2010). Any discrepancies which arose were discussed and resolved by the researchers, or were referred to the principal investigator of the study for final decision. The inclusion criteria used to evaluate the suitability of an article for this review were: (i) intervention population aged over 50, (ii) intervention population in prison, (iii) interventions supporting the social care needs of older prisoners by any professional group, (iv) intervention explicitly involving social workers or other social care staff; and (v) published in English or French. Articles were therefore included if they detailed any interventions, activities or programmes addressing social care needs in a prison setting specifically related to age-related disability or end of life. Papers were excluded if they: (i) focused on issues of ageing in prison but did not discuss specific interventions, programmes, or activities; (ii) solely focused on pharmacological or psychotherapeutic interventions; (iii) related to prisoners of war or psychiatric inpatient units; and (iv) articles related to elder abuse, fear of crime, or crimes against the elderly*.*

Three independent reviewers extracted information from the selected papers using a standardised data extraction form based on the Population, Intervention, Comparator and Outcomes (PICO) formula (Richardson, Wilson, Nishikawa, & Hayward, 1995). Taking in addition also: author(s), article date, country, type of intervention, who delivered the intervention (including prisoners), location, age of the intervention population, research design, research participants, summary of findings and study limitations. All of the studies were double extracted to check consistency.

### Quality appraisal

Hawker, Payne, Kerr, Hardey, and Powell (2002) single quality appraisal tool was used to assess all of the empirical studies, as it covered a variety of methodologies. The Critical Appraisal Skills Programme [CASP] Systematic Review Checklist was used to supplement the assessment of reviews (Critical Appraisal Skills Programme, 2017). The appraisal was conducted only on empirical studies to assess whether they met the standard norms of empirical research. The GRADE criteria (Ryan & Hill, 2016) was then adapted, by removing the initial hierarchical appraisal following Blunt (2015), and ratings from High to Low were applied to each study, based on whether there were no concerns, serious concerns or very serious concerns regarding each appraisal domain. The overall quality appraisal category assigned to each study comprised an average of the domain scores. Given the overall lack of studies in this area, no papers were excluded from synthesis on the basis of quality.

### Data analysis

The variety of papers included in this review, and the range of methodologies employed, meant that it was not feasible or appropriate to adopt a meta-analytic approach to synthesise findings (Harden & Thomas, 2005). The focus of the review was therefore more narrative, reflecting the predominantly descriptive nature of the studies returned by the search. Using the data extraction tool, each research question was answered using a narrative synthesis approach (Popay et al., 2006).

## Results

**Fig. 1:**
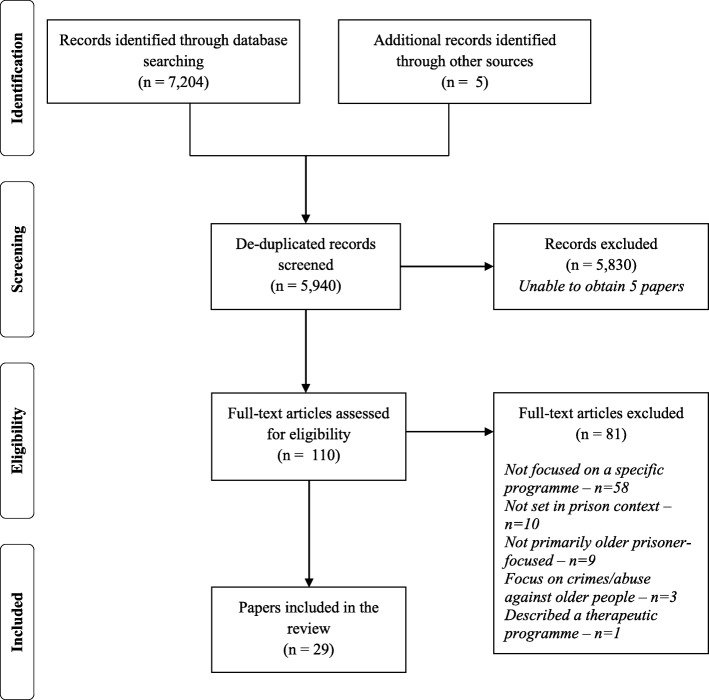
PRISMA flow diagram

Twenty-nine papers were considered to meet the criteria for inclusion in this review, and Fig. 1 depicts the stages of the screening process undertaken to reach this selection in PRISMA format:

Table 2 gives an overview of the included papers, categorised according to programme type: prison hospices, structured programmes, personal care-focused programmes, and regime (standard operational practice) and accommodation adaptations. Key aspects of each paper are presented, with main findings.

**Table 2:** Key features of the programmes described in included papers

Paper No	Author, Year, Country	Study Aim	Study type	Sample Size & Type	Intervention	Caregivers	MAIN FINDINGS for: (i) programme attendees; (ii) prisoner peer supporters (PPS); (iii) programme and prison staff; (iv) the prison; (v) costs
(I) Hospice programmes							
1	Bronstein & Wright, 2007, USA	To learn about social work & prison staff collaboration	Qualitative –telephone interviews	*n* = 14, All hospice co-ordinators	Hospice – 14 hospices, 11 states	MDT *(Psychology and business);* PPS & CVs	(i) positive care from PPSs particularly; (ii) transformational impact (self-worth increase); (iii) MDT works well; prison staff support mixed; (iv) prison management supportive, more care = better security, prison as more humane; (v) none discussed
2	Cloyes, Rosenkranz, Wold, Berry, & Supiano, 2014, USA	To explore the motivation and impact of hospice work on PPSs	Qualitative survey	*n* = 75 All PPSs – (24%) female	Hospice – 5 prisons in Louisiana, inc Angola	MDT PPS	(i) none discussed; (ii) positive impact - (re) construct identity, redemption, expressing true self, paying it forward, developing shared collective identity; (iii) none discussed; (iv) supports sense of prison as community; (v) none discussed
3	Cloyes et al., 2016, USA	To identify key factors in providing a prison hospice programme	Qualitative -interviews and observation	*n* = 43 [prison staff (*n* = 5), hospice staff (n = 14), PPS (*n* = 24)]	Hospice – Louisiana, Angola	MDT; PPS	(i) PPS care high quality; prison staff can protect, but also limit care (eg visits); (ii) critical role, free up staff; (iii) improved hospice staff practice, shared values and teamwork; some prison staff uphold hospice values; (iv) improved prison culture, supportive management – problem solve security issues; (v) none discussed
4	Cloyes et al., 2017, USA	To describe how prisoners learn to provide hospice care	Qualitative - interviews and ethnography	n = 43, [Prison officers (n = 5), hospice staff (n = 14), PPS (n = 24)]	Hospice – Louisiana, Angola	MDT; PPS	(i) none discussed; (ii) critical role, work rewarding, but stressful - grief and burn-out issues; (iii) staff support and respect PPSs, with boundaries, see them as enabling the delivery of more comprehensive care; (iv&v) none discussed
5	Hoffman & Dickinson, 2011, USA	To explore features of hospice programmes	Survey - questionnaires	n = 43 All hospice staff	Hospice – 43 programmes unknown locations	MDTs; PPS 93%, Dietician 45%;Pharmacist 21%; Family 24%; CVs 17%;Psychologist 7%	(i) none discussed; (ii) develop confidence & compassion, but emotional toll; (iii) strongly supportive programme staff; mixed support from prison staff, but lack training; (iv) strongly supportive prisoners and administration, public less so; (v) possible reduction in health care costs (less transportation and security costs), especially if use a DNR admission criteria.
6	Loeb et al., 2013, USA	To explore end-of life care values and perceptions of PPSs	Qualitative -interviews	*n* = 17 All PPSs	Hospice/ End of life care, 4 prisons in one State	MDT (*psychology support staff)* PPS (half paid)	(i) some staff lack compassion, stong bonds with PPS; (ii) transformative –non-judgmental, help self by helping others, keep out of trouble to continue with work; (iii) respect and support of PPS; (iv) improved relationships and community morale, but focus on security a barrier and some prisoners can be disparaging; (v) none discussed
7	Maull, 1991, USA	To describe the development of a prison hospice and its six-month pilot evaluation	Survey - questionnaires	n = unknown Health & prison staff; patients & PPSs	Hospice –Springfield	MDT (*Psychologist, no social worker or Dr)* PPS	(i) universally positive – PPSs helped depression, increased activeness; (ii) positive about programme, essential, work as life-enhancing; (iii) bridges are in process of being built with medical staff, with small majority of prison staff supportive but most had no opinion-apathy/lack of awareness; (iv&v) none discussed.
8	Maull, 1998, USA	To explore issues affecting hospice care delivery	Mixed: Interviews, Questionnaires; expert opinion	n = unknown. Hospice co-ordinators and staff, prison staff, PPSs	Hospice programmes in 7 states	MDT (*psychologist)*; PPS; CV	(i) fear and suspicion, some prefer to remain in mainstream prison – friends & more activities unless can get off unit, pain medication poor, trust PPSs and CVs more; (ii) key, redemptive, paying forward, (iii) staff trained to be wary of prisoners & CVs; (iv) security-care conflict, environments typically Spartan; may be seen as a death row; (v) hospice reputation as cost-effective.
9	Stone, Papadopoulos, & Kelly, 2012, UK	To examine evidence of palliative care in prisons, good practice and barriers	Integrative review	*n* = 21 5 = UK studies 16 = USA studies	Hospice /end-of-life care (UK = 5, USA-16)	MDT (*Psychiatrists, Dieticians)* PPS (majority) CV (minority)	(i) some emotional support esp. from PPSs, but some staff suspicious, and some feel they are undeserving of hospice; lack pain meds, adequate in one study, and some wariness of hospices and DNR orders; (ii) central role, emotionally rewarding, rehabilitative; (iii) suspicion of prisoners, (iv) most hospices fine, one suggested no advantages or special amenities; (v) within-prison hospices can be “cost-effective”
10	Supiano, Cloyes, & Berry, 2014, USA	To explore the impact of caring for dying prisoners on PPSs	Qualitative interviews	*n* = 36 All PPSs	Hospice – Louisiana Angola	MDT (PT, OT, dietician) PPS	(i) none discussed; (ii) work can exact overwhelming emotional toll on PPSs, sense of purpose and mutual support helps; (iii) support to PPSs from programme staff., prison staff not discussed; (iv) took years for prisoner community to trust in hospice (v) none discussed
11	Wion & Loeb, 2016, USA	To review end of life care for prisoners	Systematic review	*n* = 19 1 = UK study 18 = USA studies	Hospices/ End of life care - various	MDT (*Psychologist, dieticians, pharmacists, OTs, PTs, admin)* PPS CV	(i) good care from PPSs, promote dignity and respect, varied staff care and compassion and pain meds; (ii) transformative (increased compassion and confidence), redemptive, paying it forward, good buffer for staff; (iii) varied reports of: team cohesiveness; prison staff mixed support – security concerns, and not punitive enough; (iv) positive support from management & prisoners although some negative, prison more humane, inappropriate environments for some (buildings, equipment & comfort), lack public support; (v) seen as cost-effective.
12	Wright & Bronstein, 2007a, USA	To *explore prison hospice functions, integration in the prison and impact*	Qualitative interviews	n = 14, All hospice co-ordinators	Hospice – 14 hospices, 11 states	MDT (inc psychologist, & business) PPS	(i) positive impact, some prison staff see prisoners as undeserving; (ii) vital role, transformed, more compassionate, all positive about their role; (iii) mostly positive programme staff, unsupportive of PPSs in one hospice; prison staff mixed support; (iv) most management supportive (a couple not), more humane prison; coupled with positive media attention; (v) none discussed.
13	Wright & Bronstein, 2007b, USA	*To explore hospices integration in prisons, and staff team working*	Qualitative interviews	n = 14, All hospice co-ordinators	Hospice – 14 hospices, 11 states	MDT (dietician, psychiatrist, PTs, OTs, pharmacists, admin) PPS	(i) better, compassionate relationships with staff; (ii) vital role, increased confidence & compassion; (iii) improved staff compassion, allowed compassion to be demonstrated by staff; (iv) made prison ‘decent’ and humane; (v) none discussed
14	Yampolskaya & Winston, 2003, USA	To identify components and outcomes of prison hospice programmes	Qualitative interviews & literature review	n = unknown 10 programmes	Hospice: multiple	MDT (Psychologist, psychiatrist) PPS (most prisons) CV (2 prisons)	(i) advantage to dying with familiar people and surroundings, ‘better’ pain management; (ii) transformative and rehabilitative; (iii) none discussed; (iv) prison & hospice goals different, but sends message that prisoners can die with dignity; (v) hypothetically cost-effective – reduced hospital visits, transport, medical and staff costs, and use of DNR orders.
15	Cichowlas & Chen, 2010, USA	Description of a hospice programme	Descriptive	n/a	Hospice, Dixon, Illinois	MDT (*psychologist, psychiatrist)* PPS	Successful overall: (i) none discussed; (ii) transformative; (iii) none discussed; (iv) hospice as more institution-centred than patient-centred; (v) no additional prison funding; do use an inmate benefit fund
16	Evans, Herzog, & Tillman, 2002, USA	To describe a prison hospice programme	Service description	n/a	Hospice – Louisiana, Angola	MDT; PPS	(i) peace of mind, but mistrust staff; (ii) increase self-confidence; (iii) rewarding work for programme staff; mixed prison staff support; (iv) improved public image; prisoners supportive; (v) No extra cost (believe saves money) – healthcare redeployed; fund-raisers, outside donations.
17	Head, 2005, USA	Commentary of hospice tour by hospice experts	Descriptive	n/a	Hospice, Louisiana, Angola	MDT, PPS	(i) less scared of dying alone and in pain; (ii) dedication and transformation; (iii) none discussed; (iv) less violent, more caring prison culture, “not plush by any stretch of the imagination” (p 357); (v) no additional costs
18	Linder, Knauf, Enders, & Meyers, 2002, USA	To describe a prison hospice	Descriptive	n/a	Hospice care, Vacaville, California	MDT (*Psychiatrist)* PPS CV	(i) described as providing for all needs, peaceful place to die; (ii) cornerstone, paying it forward, may be rehabilitative; (iii) prison staff difficulty reconciling security & care; (iv) hospice transformed from a ‘snake-pit’ to respectful environment to die; (v) none discussed
19	Ratcliff & Craig, 2004, USA	Description of the GRACE project	Descriptive	n/a	Hospice −4 states	MDT PPS in two sites	(i) positive impact, with ‘exceptional’ PPS support; (ii) transformative; (iii) increase in staff morale; (iv) decline in violence and litigation; (v) cost neutral, but lack of funds limited educational activities
20	Zimmermann, 2009, USA	To describe the development of a prison hospice	Descriptive	n/a	Hospice, Connecticut	MDT PPS CV	(i) positive impact; (ii) transformed, allowed to be compassionate; (iii,iv) none discussed; (v) cost neutral, potentially cheaper – transport, PPS & CV, DNR orders and redeployed staff; training by community hospice at no cost
(II) Structured programmes							
21	Kopera-Frye et al., 2013, USA	To examine effects on prisoners, (veterans and non-veterans)	Cross-sectional standardised questionnaires	*n* = 111 Prisoners	True Grit – a structured living programme	Community Volunteers & Psychologist	(i) Increase in prisoners’ self-reported physical & mental health, and satisfaction - no significant difference between veterans and non-veterans; (ii) not applicable; (iii) none discussed; (iv) supportive management; (v) no cost due to volunteers and donations from community organisations; believe better prisoner health will reduce costs
22	Harrison, 2006, USA	To describe a programme and its impact	Descriptive	n/a	True Grit – a structured living programme	Psychologist; CVs	(i) Reduced infirmary appointments, meds & fear of dying alone; increased wellbeing, activeness & hope; (ii) not applicable; (iii) none discussed; (iv) prison-more humane, management support, better held away from medical centre; (v) No funds – donations, volunteer labour.
23	Harrison & Benedetti, 2009, USA	Description of programme	Descriptive	n/a	True Grit – a structured living programme	Psychologist; CVs	(i) accomplishment and self-esteem, may aid health, reduction in infirmary visits and medications; (ii) not applicable; (iii) supportive; (iv) management supportive; (v) negligible – donations and volunteers
24	Hodel & Sánchez, 2013, USA	Description of programme content and delivery	Descriptive	n/a	Special Needs Program for Inmate-Patients with Dementia (SNPID)	MDT (healthcare, prison staff) PPS	(i) person with dementia can function in prison; quality of life increases, behavioural problems reduce; (ii) none discussed; (iii) work is rewarding for programme staff; (iv) important to adjust environment or provide specific units; (v) None discussed
(III) Personal care-focused programmes							
25	Chow, 2002, USA	To describe the establishment of a programme	Descriptive	n/a	Nursing programme & Hospice – South Western State	Nurses; Hospice MDT; PPS	(i,ii,iii) none discussed; (iv) challenge in reconciling security and philosophy of care; (v) belief in ‘efficient and cost-effective nursing’.
26	Sannier, Danjour, & Talamon, 2011, France	To describe a service adapted for older prisoners	Descriptive	n/a	In-cell care programme, Liancourt prison	Healthcare staff	(i) increased self-respect; (ii) not applicable; (iii) staff communication to broader medical team improved quality/timeliness of health intervention; consent issues re sharing information with staff; (iv,v) none discussed.
(IV) Regime & accommodation adaptation							
27	Moll, 2013, UK	To identify and share good practice in treatment & management of prisoners with dementia	Qualitative	n = unknown (14 prisons) Prison staff, CVs, healthcare staff	Regime/accommodation adaptation, Structured programmes, Hospice: (UK = 8, USA = 4, Japan = 1, Belgium = 1)	Varied – MDT, PPS (in 10 prisons); CV	(i) prisoners’ improved mental/physical/social wellbeing at day centre & structured programme (True Grit); wing exercise & forums positive; strong PPS-prisoner relations and SNPID success; environmental change increase confidence/independence, reduce anxiety/confusion; (ii) none discussed; (iii) integration hampered by staff shortage, with PPS boosting capacity; dementia trained staff more confident; (iv) none discussed; (v) no costs presented, but specialist units and environmental change costly, voluntary sector input can be no cost or inexpensive
28	Hunsberger, 2000, USA	To describe the conversion of a mental hospital to a prison	Descriptive	n/a	Accommodation adaptation, Life Skills Program, Pennsylvania (Laurel Highland)	MDT	(i,ii) none discussed; (iii) third of prison staff are nurses so may aid the security-care conflict; (iv) management support, media attention: “a prison with compassion”; (v) $26 million conversion from mental health hospital to prison, but programme costs themselves not presented.
29	McCarthy & Rose, 2013, USA	Discussion of how States are addressing ageing prisoners	Descriptive	n/a	Regime & Accommodation adaptation, Hospice (8 states)	MDT PPS (hospice)	(i,ii,iii,iv) none discussed; (v) hope health care prison facilities will be cost-effective. Couple of prisons had similar or less costs for older prisoners than nursing homes; one hospice programme (Angola) had no extra costs; costs of specialist health unit beds in two prisons (inc Laurel Highland) were greater than for average prisoner beds.

*MDT =* Social workers, nurses, doctors, chaplains and prison staff, all else listed are in addition to this core group; PPS = Prisoner Peer Supporters; CV = Community Volunteers

The papers included predominantly came from the USA (*n* = 26), or included prisons from the USA (*n* = 28), with only one paper produced and focused exclusively on a French prison (no 26). Another paper also included prisons from the UK, Japan and Belgium (no 27). Whilst seven of the papers were not specific about the gender of the prisoners involved, all were situated fully or mostly in male prisons, with only four studies including a female prison. Further socio-demographic information was missing from the majority of papers.

### Research question one: what types of programme were available to support the social care needs of older prisoners?

The papers included in this review were primarily categorised into four types of programme: hospice, (*n* = 20; nos 1–20), structured programmes (*n* = 4, nos 21–24); personal care-focused (n = 2, nos 25–26); and regime and accommodation adaptation (*n* = 3, nos 27–29). It is of note that a couple of the papers also overlapped categories (nos 24–25,27–29).

### Hospice programmes

The prison hospice programmes provided end-of-life care, including pain management, comfort, psychological and social care, often with more flexible and frequent visits from family (nos 5,8,14,16,18), and prisoner friends (nos 5,8,15-16,18). All of the programmes described were situated within existing prison hospital or long-term care units in USA prisons only (nos 3,6,10-11,16), and were on average 2–3-bed in size (nos 3,5,8,9,18,29). The programmes were typically staffed by a core group of prison, healthcare and social care staff – who were often involved in the development and management of these programmes. One prison also included family as members of the care team (no 5).

### Structured programmes

Three structured programmes were detailed in the papers, all located in the USA: True Grit (nos21–23,27), the Special Needs Program for Inmate-Patients with Dementia (SNPID) (nos 24,27) and Living Skills (no 28). All of these involved a combination of individualised and group programmes which included: daily living skills, employment, socialising, exercise, and resettlement activities, as well as ‘treatment’ (eg substance misuse). Whilst one of the programmes was delivered off-wing by a psychologist with community volunteers (nos 21–23,27), the other two were embedded in the practices of the wings where the prisoners were accommodated, including some environmental adapations, and involved prisoner peer support assistance to prison and healthcare staff (nos 24,27–28).

### Personal care-focused programmes

Both programmes (nos 25–26) described services which supported prisoners with ADLs, with a view to maintaining independence and functioning. One was delivered by nurses and prisoner peer supporters in a healthcare unit in an American prison (no 25); the other was facilitated in-cell by care workers in a prison in France (no 26).

### Regime and accommodation adaptation

The three papers included in this category all described prison accommodation adapted for older prisoners, mostly accompanied by an alteration in the prison regime (nos 27–29). Two of the papers reported on USA prisons (nos 28–29), and the other reported on prisons in the UK, USA, Japan and Belgium (no 27).

#### Accommodation adaptation

Four papers detailed specialist accommodation for older prisoners, in the shape of separate units or wings (nos 24,27,29), or facilities (no 28), which typically provided 24-hour cover from nursing and prison staff, with some also employing peer supporters to assist older prisoners with personal care and ADLs (no 27). There were also reports of some prisons making renovations to aid prisoners ADLs, mobility and functioning, such as: adapted bathing facilities, handrails and ramps (nos 24,27).

#### Regime adaptation

Many of the prisons operated more relaxed regimes with more time unlocked through the day, a variety of leisure activities and exercise, more appropriate employment, education, library materials and activities available on- and off-wing – the latter largely facilitated by charitable organisations (no 27).

### Research question two: types of methodology and quality appraisal?

Sixteen of the papers included in this review were reports of original research or reviews (nos 1–14,21,27). The remaining 13 articles were descriptive papers (nos: 15–20,22-26,28–29). None of the studies involved experimental or quasi-experimental designs, with the majority using a qualitative methodology (*n* = 10; nos 1–4,6,10,12-14,27), a further three using questionnaires (nos 5,7, and 21 which used standardised measures), and one employing a mixed-methods design (no 8). There was also an integrative and systematic review (nos 9 and 11 respectively). Four original research studies did not report sample sizes (nos 7,8,14,27), the rest ranged between 14 and 75 participants. Excluding the reviews, only two papers sampled prisoner programme attendees (nos 7,21), although one of these did not provide any further sample details (no 7), and only one study focused on programme participants primarily (no 21). Most of the studies sampled hospice or healthcare staff (*n* = 10), with eight including prisoner supporters, and four with prison staff. None of the papers sampled prisoners’ family members or friends.

Each of the 16 original research papers were subject to a quality appraisal (summarised in Table 3). The remaining 13 descriptive papers were not fully appraised as they did not contain any methodological detail. The risk of these studies being biased was thus assumed to be high, and quality automatically categorised as low. Four of the 16 papers that were fully appraised were categorised as high quality (low risk of bias), with six categorised as of moderate quality, and six of a low quality (high risk of bias). The main issues raised by the quality appraisal were around lack of methodological detail, description of the sample, sampling and analysis, lack of clarity in presentation of the findings, and little discussion of bias. Mostly the abstract, aims and background contextual detail were clear and thorough, and the work was judged to be of value despite quality issues.

**Table 3:** Quality appraisal of included papers

Paper No	Author(s)	METHODOLOGICAL APPRAISAL SUMMARY		Quality Category
		Strengths	Limitations	
1	Bronstein & Wright	Structured and full abstract, background and aims; inclusion of interview protocol; clear data collection process; some discussion of analytic process and triangulation; secured appropriate ethical approval; structured results section	Questionable methodological appropriateness*;* interviews not taped, but used quotes; lack of detail of sampling, informed consent & data analysis; conclusions made about prisoners limited by not talking to any; limitations & biases not discussed	LOW
2	Cloyes, Rosenkranz, Wold, et al	Structured and full abstract, background and aims; full data analysis description; secured ethical permission and described informed consent process; clear presentation of results	Lack of explanation of method; patchy socio-demographics, although discussed; no reflections on researcher bias	HIGH
3	Cloyes, Rosenkranz, Berry et al	Structured and full abstract, background and aims; interviews recorded; fairly large sample size; full data analysis description, validation & triangulation; ethics approval; thorough results section	Lack of detail about interviews; no interviews with prisoner patients; no socio-demographic information; no description of informed consent process; assume programme is effective, no evidence presented; bias not discussed	MODERATE
4	Cloyes, Rosenkranz, Supiano, et al	Structured and full abstract, background and aims; taped interviews; method appropriate; quite large sample size; full data analysis description, validation & triangulation; ethics approval; full discussion of study implications	Lack of detail about the interviews; did not interview prisoner patients; no socio-demographic detail presented; bias not discussed; results about prisoner volunteers contained no detail from them and no quotes throughout; opinion presented as fact	MODERATE
5	Hoffman & Dickinson	Clear and informative abstract and introduction; sampling strategy detailed, and good size and breadth, with high response rate	Aims not wholly clear; methodology detail scant, esp. on surveys used; no socio-demographic, data analysis, ethics or bias information; findings lack clarity; opinions stated as fact	LOW
6	Loeb, Hollenbeak, et al	Structured and full abstract, background, aims, methods, sampling, data analysis and findings; presented discussion guide; thorough discussion of ethics and bias	Quite small sample size; interviews not taped but used quotes; prisoner patients not sampled	HIGH
7	Maull	Report of one of the first in-prison hospice programmes, which influenced their development across the USA.	Lack of evaluation detail in abstract, lack of evidence for background; lack of information on methods, sampling, analysis, ethics and bias, and few findings presented.	LOW
8	Maull	Fairly comprehensive background, guidelines resonate with later research, discussion of implications.	Lack of detail in abstract, literature review used only one database but information not synthesised, vague aim, inadequate method, sampling, data analysis, ethics & bias and findings.	LOW
9	Stone, Papadopoulos, et al	Clear abstract and aims and method guideline; value as first review of hospices published	Justification of UK–USA comparison weak; some background lacking; no quality appraisal; data sampling confusing; search strategy not exhaustive; data extraction unclear; triangulation unmentioned; unclear results; conclusions overstated	LOW
10	Supiano, Cloyes & Berry	Clear abstract and aims, full background, taped interviews, included interview guide, clear sampling, full data analysis description, socio-demographic and ethics information, clear results, discussion of limitations and transferability issues	Full confidentiality could not be guaranteed, was discussed as a limitation; hospice presented as ‘thriving’ with no evidence in support of that assertion, and ‘recent’ even though in existence for 16 years.	HIGH
11	Wion & Loeb	Clear abstract, methodological guidelines, quality appraisal & extraction method, as well as validation and triangulation; results detailed and easy to follow; discussed implications & limitations	Background brief, 6 research questions; searched 5 databases using 4 search terms only; author bias issue not fully justified; results not always well synthesised & very lengthy	MODERATE
12	Wright & Bronstein a	Structured and full abstract, background and aims; discussion of bias affecting result; ethics permission obtained; structured findings	Only sampled hospice leads; no information on interview guide topics; sampling strategy not apparently comprehensive; brief analysis, did not tape interviews; no informed consent discussion; results not always synthesised; extensive quotes used – but not verbatim transcripts; v similar to previous study	MODERATE
13	Wright & Bronstein b	Structured and full abstract, background and aims; discussion of bias affecting result; question used was presented; ethic approval granted	Only sampled hospice leads; lack of sampling and analysis detail; did not tape interviews but presented ‘quotes’; no informed consent process described; findings brief relative to Introduction; results not always synthesised; similar results to previous work	MODERATE
14	Yampolskaya & Winston	Fairly comprehensive abstract and background; attempt to contact ‘all’ prison hospices; findings have proved influential, especially the components identified	Some missing info from abstract, introduction lacked references; lack of methodological and sampling information; no socio-demographics; very basic analytic information, none on ethics nor bias; findings confused and lacked detail	LOW
15	Cichowlas & Chen	No methodology to appraise		LOW
16	Evans, Herzog et al	No methodology to appraise		LOW
17	Head, 2005	No methodology to appraise		LOW
18	Linder, Knauf et al	No methodology to appraise		LOW
19	Ratcliff & Craig	No methodology to appraise		LOW
20	Zimmermann	No methodology to appraise		LOW
21	Kopera-Frye, Harrison, et al	Mostly full abstract, background and aims; good description of surveys (some standardised), data collection and sample with socio-demographics and response rate; ethics and informed consent discussed; detailed findings; sampled prisoners	Not all assertions for background were evidenced; some lack of data analysis detail in methods section, especially qualitative; no bias discussion; results not presented in easiest way to follow, especially qualitative	HIGH
22	Harrison, 2006	No methodology to appraise		LOW
23	Harrison & Benedetti	No methodology to appraise		LOW
24	Hodel & Sanchez	No methodology to appraise		LOW
25	Chow	No methodology to appraise		LOW
26	Sannier, Danjour et al	No methodology to appraise		LOW
27	Moll	Mostly full abstract, full background details on areas asked about in survey; some methodological and sample detail; detailed findings and recommendations	Lack of methodology detail, data collection, sampling strategy, and participant numbers; no prisoners sampled; analysis technique, informed consent & biases not presented; very difficult to follow findings which are mostly unsynthesised	MODERATE
28	Hunsberger	No methodology to appraise		LOW
29	McCarthy & Rose	No methodology to appraise		LOW

### Research question three: what were the programme outcomes?

The papers included in this review described a number of ways in which older prisoner-focused programmes had an impact on prisoner programme attendees, prisoner peer supporters, programme and prison staff, the prison and wider prisoner community, the community outside, and costs. The section will present outcomes in each of these areas for each programme type, where reported.

### Prisoner programme attendees

*Hospice programmes -* five papers did not discuss the impact of the hospice on patients (nos 2,4-5,10,15), with the remaining papers reporting a mixed experience for the prisoners. Some papers suggested staff provided compassionate care (nos 3,11,13), with others reporting a lack of staff compassion (nos 3,6,9,11,12), and of prisoner mistrust of staff and the hospice overall (nos 8,9,16), including the quality of pain management which reportedly varied from adequate to poor (nos 5,8-9,11,14). All of the papers reported positive experiences of prisoner peer support (nos 1,3,5,6,8-9,11,13-14,19), including the one paper which actually sampled prisoner-patients (no 7), and many of a lessening in prisoners’ fear and likelihood of dying alone (nos 10, 14,16,17,18), although one reported less access to friends and prison activities (no 8).

*Structured programmes –* positive outcomes were reported regarding prisoners’ physical, mental and social wellbeing for the True Grit and SNPID programmes (nos 21–24,27), although only one paper sampled prisoner attendees (no 21). These included a reduction in medication use, medical appointments (nos 22–23) and behavioural problems (nos 24); and an increase in activeness (nos 22–23), quality of life (no 24), and confidence (nos 23). The Life Skills programme did not report attendee outcomes (no 28).

*Personal care-focused programmes –* one paper did not report outcomes (no 25), the other suggested enhanced prisoner-patients’ self-respect (no 26). *Regime/accommodation adaptations –* there were no findings discussed by two papers (nos 28–29). The remaining paper suggested that adaptations could improve wellbeing (no 27).

### Prisoner peer supporters

*Hospice programmes –* many of the papers explicitly focused on prisoner peer supporters, describing them as key to operations (nos 3–4,7-9,12-13,18). In addition, the work reportedly had a transformative effect on their self-confidence (nos 1,5,11,13,16), sense of compassion and community (nos 2–3,5,6,11,13,20), increasing opportunities for redemption and rehabilitation (nos 1–2,6,9,13–14), and a predicted reduction in recidivism (nos 7–8,13). Whilst, the prisoner peer supporters were reported to find the work rewarding (nos 4–5,9–10), some also reported grief and burnout (nos 4–5,10), exacerbated by inadequate training and supervision (no 11).

*Structured programmes -* peer supporters were deemed successful within the SNPID programme (no 27), and reportedly found the work rewarding (no 24). There were no outcomes discussed (no 28) nor peer supporters employed (nos 21–23) in the other two programmes.

### Programme and wider prison staff

*Hospice programmes –* programme staff largely reported positive experiences of hospice work, finding it rewarding (nos 3,11-13,16) and impacting helpfully on morale and intra- and inter-team relationships (nos 1,5,11,19), including with prisoner peer supporters (nos 3–4,6,11,13-14,16). However, there were also reports of some programme staff being resistant to working with prisoner peer supporters (nos 8–9,12), and of conflict between programme and wider prison staff due to a clash of priorities between care and security (nos 1,3,11,14). Prison staff were described as more mixed in their support of hospices (nos 1,4-6,8-9,12–13), particularly with regard to prisoner peer supporters, however it was also suggested that this shifted across time and with greater exposure to programmes (nos 13,19). Staff outcomes were not discussed in five papers (nos 2,14-15,17,20).

*Structured programmes –* discussion of staff experiences were minimal, with none in two papers (nos 21–22). One paper did suggest that prison staff were supportive of one programme (no 23), and that programme staff found the work rewarding in another (no 24).

*Personal care-focused programmes –*communication between programme and health staff reportedly improved in one programme (no 26). There was no discussion regarding staff impact in the other (no 25).

*Regime and accommodation adaptation –* two papers reported that co-working between prison staff and staff from other prison departments such as health, was hampered by low numbers – which negatively affected making adaptations, although this was seemingly ameliorated somewhat by training (nos 27–28), such as health awareness training for prison staff (no 27). One paper did not discuss outcomes in this area (no 29).

### The wider prisoner community and prison infrastructure

*Hospice programmes -* these programmes appeared to garner support from the wider prisoner community (nos 5,11,16) and prison management (nos 1,3,5,11–12), with hospice-containing prisons perceived as more decent and humane, and linked with lower levels of violence (nos 1,2,3,6,11,12,13,17,19). However, some reported that prisoners viewed hospices suspiciously, as de facto ‘death rows’ (nos 6,8,10–11). There was also a report of mixed managerial support (no 12), and of a clash of philosophies between security and care (nos 3,6,8,14–15), possibly manifesting in hospice environments of “pervasive drabness” (nos 8, p 65, 9,11,17). Some hospices were deemed inappropriate, lacking resources and equipment (nos 8–9,11), and others were found to be comfortable (nos 9,11,17).

*Structured programmes –* two programmes highlighted support provided by prison management (nos 21–23,28), with one programme perceived as promoting a more humane prison (no 22). Two papers also reported the importance of programme location, including environmental adjustments or specialised units for prisoners with dementia (no 24) and situating programmes away from medical wings (with some seen as “death row”, no 22). *Regime and accommodation adaptation –* one paper reported restrictions to accommodation adaptation due to ageing buildings and budget limitations (no 27).

### The ‘outside’ community

There were reports of positive media attention for one prison hospice (no 12), and a prison which had undergone extensive renovations (no 28). Where reported, there appeared to be mixed public support for prison hospices, with suggestions that their existence improved one prisons’ public image (no 16), but a lack of public support associated with others (nos 6,11).

### Costs

*Hospice programmes -* none of the included papers conducted cost-effectiveness studies, and many presented no cost information (nos 1–4,6,10,12-13,18). Some suggested hospices could be cost-effective (nos 8–9,11), or cost-neutral (nos 15–17,19–20) due to staff redeployment, volunteers, free training, and external donations (nos 15–16,20). A couple of papers suggested that hospices could save prisons money, with reduced transport, security and healthcare costs (nos 5,14,16,20).

*Structured programmes -* there were no cost-effectiveness studies, and no costs presented for two programmes (nos 24,28). The True Grit programme reported that there were no additional costs associated with running the programme, as it relied on redeployed or voluntary labour, and donations (nos 21–23), with a predicted overall reduction in costs as prisoner health improved (no 21). *Regime and accommodation adaptations -* no papers detailed cost-effectiveness, but all provided cost information. Adaptations or specialist units were reportedly costly (nos 27,29). For example, beds at one dementia-dedicated unit were double that of the average, although it was suggested that these costs could become cost-effective long-term (no 29).

## Discussion

This integrative review found 29 papers which described programmes that supported the social care needs of older prisoners, most of which were from the USA and described hospice programmes, thus there were more reported outcomes for these. However, there were also papers describing structured programmes, personal care-focused services and regime and accommodation adaptations, including two from other (high income) countries. Overall, the programmes were reported to have a generally positive impact, with the transformative effect upon the prison overall and prisoner peer supporters most frequently reported, and peer care particularly commended for the hospice programmes. There were more mixed reports of staff care, community support, and team functioning in the hospice programmes, and some difficulties arising from blending care and security concerns. Some positive impacts on prisoner’s wellbeing were reported by all programme types, although most prominently for the structured programmes. All programmes were hypothesised to be cost-effective or cost-neutral in the long-term. However, the evidence found in this review should be interpreted with caution, given the low quality of the majority of the reviewed papers, a marked lack of experimental or quasi-experimental effectiveness or cost-effectiveness studies, together with the lack of participation of prisoner programme attendees in all but one of the papers.

The limited evidence base appears to arise from a range of barriers including difficulties gaining consistent access to prisoners with often multiple layers of bureaucracy to negotiate, and prisoner transfers around the prison system (Ahalt, Haney, Kinner, & Williams, 2018; Apa et al., 2012; Lučić-Ćatić, 2011). Additionally, spending on prison research is relatively low in many countries with less than 0.1% allocated to criminal justice health research in Canada and the USA for example (Ahalt, Bolano, Wang, & Williams, 2015; Kouyoumdjian, McIsaac, Foran, & Matheson, 2017). Against a backdrop of overcrowding, and escalating violence in many prison systems internationally (MacDonald, 2018; Rope & Sheahan, 2018), the lack of attention may also be due to the lack of ‘noise’ generated by older prisoners who typically reoffend less and pose fewer control problems than their younger counterparts (Ministry of Justice, 2018b; Omolade, 2014; Psick, Simon, Brown, & Ahalt, 2017).

Perhaps unsurprisingly few, if any, countries have a comprehensive policy or strategy focused on the growing numbers of older prisoners despite their clear vulnerabilities and costly care needs (Atabay, 2009; Her Majesty’s Inspectorate of Prisons, 2016; Williams et al., 2012), a situation which has been described as a “human-made disaster” (Maschi, Leibowitz, Rees, & Pappacena, 2016, p 167). In England and Wales, parliamentary inquiries, inspection bodies and prison charities have called for such a strategy to no avail (Her Majesty’s Inspectorate of Prisons, 2004, 2016; Justice Committee, 2013; Prisons & Probation Ombudsman, 2016, 2017). Although the policy-practice-research relationship is complex, these calls are likely to be strengthened by more robust research, particularly cost-effectiveness studies (Qureshi, 2002; Whiteford & Weissman, 2017). However, it is also of note that the establishment of the SNPID programme (Hodel & Sánchez, 2013) was in response to prisoner litigation regarding the inadequacy of care for those with cognitive impairments.

The extent to which the existing evidence base can prompt policy and practice shift in this area is limited, and highlights the embryonic stage that research into supporting the social care needs of older prisoners is at in its ‘evidence journey’ (Nutley, Powell, & Davies, 2013). However, it is also of note that there are likely many effective initiatives taking place within prisons which have not yet been reported on, which gives a further rationale for investment in research and evaluation in partnership with the prison staff, prisoners and the external (often charitable) organisations who developed them. Given the vulnerability of this prisoner group, further research with the following foci may be considered an ethical imperative:

- *Identifying older prisoners’ social care needs –* important to establish for the development of policy and in commissioning services. This is also consistent with recent government guidance in one country (Munday, Leaman, & O’Moore, 2017)
- *End-of-life care and hospices* - although compassionate or early release may appear to be the most obvious route for prisoners reaching the end of their lives or with significant health and social care needs such as dementia, in many high income countries it is used sparingly for fear of public censure (Justice Committee, 2013; Loeb, Penrod, McGhan, Kitt-Lewis, & Hollenbeak, 2014). Therefore, evaluations of various end of life options could be useful, potentially building upon the USA-based hospice work, but including cell-based and community transfer options as well.
- *Personal care* – this could include the development and evaluation of a model of practice to reflect the main way that the social care needs of prisoners are likely to be assessed and attended (Tucker et al., 2017).
- *Structured programmes* – the building and evaluation of programmes of activities for prisoners, potentially by using successful community programmes adapted for prison and for post-release reintegration – which is a particularly under-researched area regarding older prisoners (Cooney & Braggins, 2010; Kamigaki & Yokotani, 2014).
- *Regime and accommodation adaptation* - there has been considerable debate around the use of segregated wings or units for older prisoners (Doron & Love, 2013; Lee et al., 2016; Wangmo, Handtke, Bretschneider, & Elger, 2017). Partial segregation, whereby prisoners live in a separate unit adapted for older prisoner needs in terms of rules, activities and environment, but mix with other prisoners if desired when accessing prison-wide activities and services, has been advocated more recently (Kerbs, Jolley, & Kanaboshi, 2015; Wangmo et al., 2017). However, this debate would benefit from further evaluation.
- *Team working -* given the different staff and prisoner groupings involved in delivering social care in prisons, and associated security-care philosophy clashes, research exploring team working, and the negotiation and resolution of these tensions would be useful. Many of the programmes employed prisoners, but robust evaluations of peer support for older prisoners and their co-working with staff are lacking (Stewart & Edmond, 2017).
- *Prisoner involvement and participation* - there was a striking lack of prisoner programme attendees included in the samples of the papers under review. As well as participation, future research would also likely be strengthened by the involvement of prisoners throughout the development of the research from conception to dissemination, in line with good research practice (INVOLVE, 2012).
- *Family and other carer involvement* – older prisoners’ loss of family contact has been reported as a social care need, and has been linked with a range of outcomes including rates of suicide, reoffending and post-release reintegration (Farmer, 2017; The Howard League for Penal Reform, 2016). Although not an explicit focus of any of the programmes, it would seem beneficial to develop and evaluate programmes aimed at maintaining or re-establishing older prisoners’ family links and which help prisoners’ families cope with the deleterious impact that imprisonment has on them (Breen, 2008). In addition, families and other carers typically provide large amounts of informal care to older adults in the community (Verbeek-Oudijk, Woittiez, Eggink, & Putnam, 2014), but options to involve families further in supporting prisoners, particularly in transition back to the community, are under-explored.
- *Protected characteristics* - most of the papers included were focused on male prisoners, while research has suggested that the size and type of support needs of older female prisoners differ (Aday & Krabill, 2012; Trotter & Baidawi, 2015). There was also a lack of socio-demographic detail available regarding other protected characteristics (such as race, religion and sexuality), and exploring the intersectionality of these characteristics regarding older prisoners, will be particularly important avenues for future research.

This is a systematic integrative review which was rigorously conducted and reported according to established guidelines. The review nevertheless included studies with a range of methodologies, not appraised nor synthesised according to a hierarchy of evidence (following Blunt, 2015), which diverges from typical systematic review guidelines. This was partly in response to the sparseness of the research available. Additionally, adhering to a hierarchy of evidence which explicitly promotes positivist research over other research forms was not felt to be a tenable stance given the papers available (Blunt, 2015; Mallett, Hagen-Zanker, Slater, & Duvendack, 2012). The heterogeneity of the programmes identified by the review made comparisons between them difficult. Additionally, adopting a narrative synthesis approach has the potential to introduce an element of bias in reporting, although the use of two reviewers to extract data using a structured tool was designed to provide some counterbalance to potential biases.

All of the studies included were from higher income countries, and most were either from or included, the USA. The papers reflect the “northern epistemic hegemony” (Aas, 2012) typical in many fields of research and this, together with the penal outlier status of the USA (Lacey, Soskice, & Hope, 2017), means that generalising the review conclusions and implications beyond the USA in particular, and higher income countries in general, would need to be done with care.

## Conclusion

This review detailed programmes which supported older prisoners’ social care needs, including hospice and structured programmes, personal care-focused services and regime and accommodation adaptations. Whilst the papers presented largely positive results regarding prisoner peer supporters and the wider prison, there were mixed results for staff. Additionally, whilst there were positive claims made about the impact on the prisoners attending the programmes, only two papers actually sampled those prisoners. This together with the generally low quality of the papers, and lack of any experimental effectiveness studies, to some extent limits their utility for policy and practice. There is a clear need for more robust effectiveness and cost-effectiveness studies to better support the development of social care for older prisoners at individual, policy and practice levels.

## Additional files

Additional file 1: Completed PRISMA 2009 Checklist (DOCX 17 kb)

Additional file 2: Example Search Strategy (DOCX 13 kb)

## Abbreviations

ADL: Activities of Daily Living
CASP: The Critical Appraisal Skills Programme
HMIP: Her Majesty’s Inspectorate of Prisons
MDT: Multi-Disciplinary Team
MOJ: Ministry of Justice
SCIE: Social Care Institute for Excellence
SNPID: Special Needs Program for Inmate-Patients with Dementia

## Footnotes

1 The age cut-off for ‘older prisoner’ varies across the world, from 50, 55, 60, but is typically set at least 10 years younger than the general population, as prisoners are thought to age more rapidly due to more chaotic lifestyles, less healthcare access, substance misuse, and the stress of imprisonment (see Williams, Stern, Mellow, Safer, & Greifinger, 2012, for further discussion).
