# Supplementary material for: A systematic integrative review of programmes addressing the social care needs of older prisoners
Source: Health Justice. 2019 May 27;7:9. doi: 10.1186/s40352-019-0090-0 (PMC6717991; doi:10.1186/s40352-019-0090-0)
Supplement: Supplementary file 2 — Example Search Strategy (DOCX 13 kb) [file 40352_2019_90_MOESM2_ESM.docx]

**ADDITIONAL FILE 2: EXAMPLE SEARCH STRATEGY**

MEDLINE

1. exp PRISONS/ or exp PRISONERS/ or prisoner*.ti,ab.

2. exp CRIMINALS/

3. (inmate* or convict* or criminal*).ti,ab.

4. (secure adj2 (unit or units or facility or facilities or institution* or centre* or center*)).ti,ab.

5. (correctional adj2 (units or unit or facility or facilities or institution* or centre* or center*)).ti,ab.

6. (jail* or penitentiar* or gaol*).ti,ab.

7. felon*.ti,ab.

8. or/1-7

9. (peer adj4 (support* or help* or interven* or advoca*)).ti,ab.

10. (buddy* or buddies or friend* or "cell mate*" or mentor* or be-friend* or befriend* or "lay person*" or volunteer* or voluntar*).ti,ab.

11. ("insider*" or "listener*").ti,ab.

12. exp PEER GROUP/

13. or/9-12

14. (chronic adj2 (illness* or disease* or condition*)).ti,ab.

15. exp CHRONIC DISEASE/

16. exp PARKINSON DISEASE/ or exp ALZHEIMER DISEASE/

17. (age adj5 degenerat* disorder*).ti,ab.

18. dementia.ti,ab. or exp DEMENTIA/

19. "mobility disorder*".ti,ab.

20. exp MOBILITY LIMITATION/

21. (mobil* adj3 (limit* or difficult)).ti,ab.

22. deaf*.ti,ab. or exp DEAFNESS/ or exp HEARING DISORDERS/

23. (hear* adj3 (difficult* or limit* or disorder* or reduce*)).ti,ab.

24. ((eye* or vision* or blind* or sight*) adj3 (disorder* or limit* or reduced* or impair* or difficult*)).ti,ab.

25. exp BLINDNESS/ or exp VISION DISORDERS/

26. glaucoma.ti,ab.

27. macular degeneration.ti,ab. or exp MACULAR DEGENERATION/

28. exp MEMORY DISORDERS/

29. "memory disorder*".ti,ab.

30. exp COGNITION DISORDERS/

31. "cognition disorder*".ti,ab.

32. exp COMORBIDITY/ or co-morbidity.ti,ab.

33. exp FRAIL ELDERLY/

34. "frail elderly".ti,ab.

35. exp RIGHT TO DIE/

36. "right to die".ti,ab.

37. exp HEARING LOSS/

38. "hearing los*".ti,ab.

39. exp PALLIATIVE CARE/

40. palliative.ti,ab.

41. exp TERMINAL CARE/

42. (terminal* adj2 (car* or nurs* or ill*)).ti,ab.

43. "age related degenerative disorder*".ti,ab.

44. cancer*.ti,ab.

45. exp NEOPLASMS/

46. (disabilit* or disabled).ti,ab.

47. exp DISABLED PERSONS/

48. or/14-47

49. "mobility disorder*".ti,ab.

50. exp MOBILITY LIMITATION/

51. (mobil* adj3 (limit* or difficult)).ti,ab.

52. (independen* adj3 liv*).ti,ab.

53. exp "Activities of Daily Living"/ or exp Independent Living/

54. ((self or personal) adj5 (care or manage$)).ti,ab.

55. (dressing or feeding or eating or toilet$ or bathing or mobil$).ti,ab.

56. (social adj5 (activit$ or function$ or support$ or skill$ or adjust$ or behavio?r or facilitat$)).ti,ab.

57. (activities of daily living or adl$ or eadl$).ti,ab.

58. or/49-57

59. ((nurs* or care or caring or support*) adj5 (role* or position* or relationship* or friend*)).ti,ab.

60. 13 or 58 or 59

61. 8 and 48 and 60

62. 8 and 48

63. limit 62 to ("all aged (65 and over)" or "aged (80 and over)")

64. 61 or 63
